# Is the “July Effect” Real? Pediatric Trainee Reported Medical Errors and Adverse Events

**DOI:** 10.1097/pq9.0000000000000018

**Published:** 2017-03-14

**Authors:** Ankoor Y. Shah, Andrew Abreo, Nicole Akar-Ghibril, Rebecca F. Cady, Rahul K. Shah

**Affiliations:** From the * Department of General and Community Pediatrics, Children’s National Health System, Washington, D.C.; † Division of Allergy, Pulmonary, and Critical Care, Vanderbilt University Medical Center, Nashville, Tenn.; ‡ Department of Pediatrics, University of Miami, Miami, Fla.; §Division of Risk Management, Children’s National Health System, Washington, D.C.; and ||Division of Quality and Safety, Children’s National Health System, Washington, D.C.

## Abstract

**Introduction:**

The “July Effect” suggests an increase in patient adverse events in July compared with other months due to the introduction of new providers throughout the training continuum. The aim of this initiative was to analyze reported pediatric trainee medical errors from May through September 2015 at a tertiary care free-standing academic children’s hospital to determine if there were more reported medical errors and more adverse events from those errors in July.

**Methods:**

An error surveillance system is used to report and track near misses, adverse events, and medical errors. Three of the authors reviewed each report, which was electronically collected in the institution during the time period of interest. The reported medical error incidence per 1,000 trainee-days was compared against those in July for a significant difference.

**Results:**

There are a total of 282 trainees (86 pediatric residents, 81 nonpediatric residents, and 115 fellows) who are clinically active in the hospital at any given month. Pediatric residents had more reported medical errors in July (31) compared with May (16; *P* = 0.015), June (16; *P* = 0.019), and August (19; *P* = 0.046). There was no significant difference in the number of adverse events from reported medical errors by trainees in July (7) compared with May (5), June (8), August (4), or September (8; *P* > 0.2).

**Conclusion:**

In this single-center evaluation, there is an increase in reported medical errors involving pediatric residents in July compared with the months surrounding July. However, there is no difference in numbers of adverse events from those errors between these months.

## INTRODUCTION

In a ritualistic manner, the summer in the United States health care marks a major milestone in academic teaching hospitals where there is a planned turnover of physicians in training. In July, new physicians, having just completed medical school, begin their next level of training. Furthermore, other trainees either advance to the next year of training with added responsibility or graduate to begin their professional careers. As a result of this large turnover, concern has been raised regarding the quality of patient care during this predictable transition. The “July Effect” suggests increased patient adverse events and detrimental outcomes in July compared with other months presumably due to medical errors made by an influx of trainees inexperienced in their new roles.^1^ This has gained prominence in the minds of the general public due to frequent reports in the lay media.^2–5^

Existing peer-reviewed literature on this topic has been mixed and generally focused on adult mortality data. The assumption is that trainee medical errors are the “cause” for an uptick in untoward events.^1,6–11^ Systematic reviews have noted changes in other outcome measures besides mortality that also occur in July, such as a decrease in efficiency and increase in morbidity.^12^

Studies have suggested that pediatric adverse events, specifically to medication errors, are between 1.9 and 2.9 per 100 discharges.^13^ However, an evaluation at 2 pediatric academic teaching institutions showed a medication error rate of 55 per 100 admissions.^14^ Exactly how to determine medical errors within a hospital can be difficult. A voluntary error reporting system is a common tactic used to identify medical errors. Research shows that these systems can provide useful information regarding latent errors that lead to active errors and adverse events but do not truly provide an overall prevalence of total medical errors.^15^ Successful voluntary reporting systems are nonpunitive, confidential, independent, expertly analyzed, timely, systems-oriented, and responsive.^16^

At Children’s National Health System, each incident report is read by the Chief Risk Officer, Chief Quality and Safety Officer, Director of Clinical Risk Management, and the Director of Patient Safety. The potential for a medical error, adverse event, or patient harm is determined, and the need for cause-analysis is decided. Notable events are discussed at a weekly Executive Safety huddle with a team comprising the executive leadership of the hospital. On a biweekly basis, the President and Chief Executive Officer of the hospital has a discussion with this team regarding the recent safety and grievance events. Afterward, system-based actions are pursued to prevent future events.

There is minimal research regarding the “July Effect” in pediatrics. Published studies have focused on specific pediatric specialties rather than broadly across pediatric institutions.^17,18^ Differences in pediatric patient care and safety, pediatric illnesses, and resident duty hour restrictions in the last decade suggest the need for a contemporary “July Effect” evaluation in pediatrics.^19^ Most importantly, to truly understand the premise of a “July Effect,” we must determine if medical errors by trainees translate into adverse patient events. The aim of this initiative was to analyze medical errors reported by pediatric trainee (resident and fellow) from May through September 2015 at a free-standing tertiary care academic children’s hospital to identify if there are more reported medical errors and adverse events from those errors in July.

## METHODS

Incident reports were reviewed from May 1, 2015, through September 30, 2015. The institution broadly encourages the reporting of incidents and events, including medical errors that were a deviation from expected care into our electronic surveillance system RL Solutions (RL Solutions, Toronto, ON, Canada). Every 18 months, an institutional Safety Attitude Questionnaire is administered to assess the safety culture, including willingness to submit incident reports. All staff members of the hospital, including physician staff, are encouraged to submit reports. Reports can be submitted anonymously, by a witness to the event, or by the involved party. All incident reports that were submitted from May 1, 2015, through September 30, 2015, were reviewed in a blinded fashion. Information contained in each report included a description of the event, departments involved, and type of personnel involved (i.e., pediatric residents, nurse, and so on). The date and patient identifiers were removed before the authors received the reports. Significant institutional efforts have been in progress to improve the number of incident reports, with a goal of 10,000 reports for the fiscal year 2016; however, during the period of this initiative there were about 5,000 annual reports.

Three reviewers (authors: A.A., A.S., and N.A.G.) independently reviewed each incident report. The reviewers completed an internal safety and quality improvement curriculum before this evaluation. The reviewers determined if a trainee contributed to the incident. The Institute of Medicine definition of a medical error was used for this project, specifically as “a failure of a planned action to be completed as intended or the use of a wrong plan to achieve an aim.”^20^ Deviations from the process of care may or may not cause harm to the patient.^21^ A trainee was defined as a pediatric resident, fellow, or nonpediatric resident (i.e., surgical, anesthesiology, emergency medicine, and so on). The reviewers determined the incident was a trainee medical error if a trainee’s action or omission of an action contributed to the medical error. The reviewers also determined if an adverse patient event occurred due to the incident. An adverse patient event was defined, per the Institute of Medicine, as “an injury caused by medical management rather than by the underlying disease or condition of the patient.”^20^ In this evaluation, the trainee medical error led to these adverse patient events.

An incident was included in the project if all 3 reviewers agreed on trainee involvement. If disagreement occurred, the incident was discussed between the 3 reviewers to arrive at a consensus decision.

The number of clinically active trainees at the hospital per month was provided by the hospital’s Office of Graduate Medical Education. Trainees working at other hospitals, on nondirect patient care–related electives, research electives, or vacation were not considered clinically active and were not included. All incidents were categorized by month and stratified by both the type of trainee and presence of an adverse event. Comparative analysis was done between reported trainee medical errors and trainee-contributed patient adverse events from those medical errors in July against those in May, June, August, or September. Reported trainee medical errors and adverse events were converted to incidence per 1,000 trainee-days for the purpose of conducting comparative analysis. Trainee-days were calculated by multiplying the number of days in the month by the number of clinically active trainees for that month. Fisher’s exact test was used for statistical analysis. An alpha level of 0.05 was considered significant. All data analyses were completed using SAS version 9.2 (SAS Institute Inc., Cary, NC) and the Centers for Disease Control and Prevention OpenEpi application.^22^ This was a project undertaken as a quality and safety improvement initiative at Children’s National Health System and did not constitute human subjects research.

## RESULTS

**Fig. 1.**
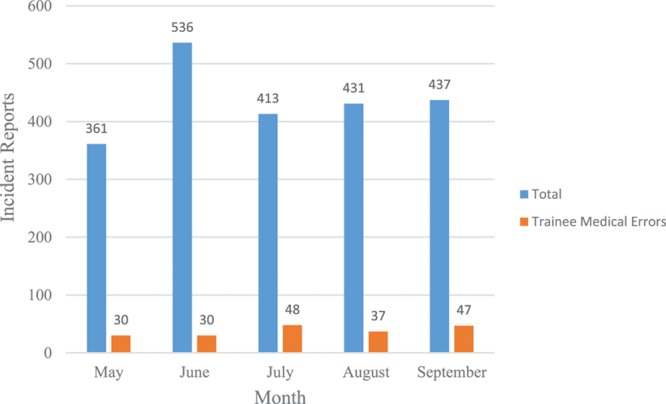
Total number of incident reports and reported trainee medical errors by month.

There were a total of 362 trainees employed at the children’s hospital with 282 (86 pediatric residents, 81 nonpediatric residents, and 115 fellows) clinically active trainees during any given month of the evaluation period. July had the second lowest number of incidents filed with 413 compared with the lowest in May (361) and highest in June (536; Fig. 1). The Fall 2015 Safety Attitude Survey at the institution had 87% of respondents strongly agree to the following statement: “I am encouraged by others in this work setting to report any safety concerns I have.”

The interreviewer agreement for pediatric trainee–reported medical errors as well as adverse events from those errors were both high (κ = 0.73 and κ = 0.79, respectively). In July, a total of 48 trainee medical errors or 5.49 medical errors per 1,000 trainee-days were reported (Table 1). This is significantly higher than the 30 reported medical errors each in May (3.43; *P* = 0.021) and June (3.55; *P* = 0.030). Similar to July, 47 medical errors were reported in September (5.56; *P* = 0.46). Thirty-one pediatric resident medical errors or 11.63 per 1,000 trainee-days were reported in July. These were significantly higher than 16 in May (6.00; *P* = 0.015), 16 in June (6.20; *P* = 0.019), and 19 in August (7.13; *P* = 0.046), but not significantly higher than 23 in September (8.91; *P* = 0.17). Fellows had 5 medical errors reported in the incident reporting system in July (1.40), which was the fewest compared with other months, although not a statistically significant difference. Of total incident reports by month, July and September had a higher percentage of pediatric trainee–reported medical errors (11.6% and 10.6%, respectively) compared with May (8.3%), June (5.6%), and August (8.6%; *P* = 0.026).

**Table 1.**
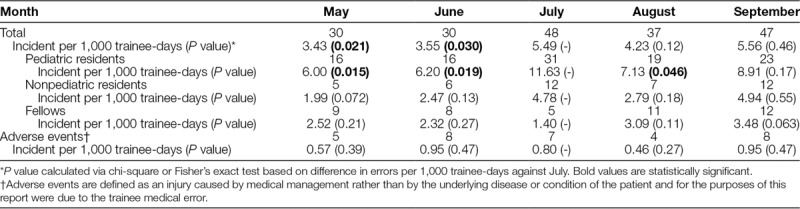
Number of Trainee Medical Errors and Patient Adverse Events by Month Compared with July

Less than 15% of reported trainee medical errors resulted in an adverse patient event. There was no difference between those 7 adverse patient events in July compared with 5 in May (0.57; *P* = 0.39), 8 in June (0.95; *P* = 0.47), 4 in August (0.46; *P* = 0.27), or 8 in September (0.95; *P* = 0.47).

## DISCUSSION

This evaluation is unique in that it is the first to directly evaluate reported trainee medical errors and their link to adverse patient events in pediatric patients in July. Our analysis showed a significant increase in reported trainee medical errors in July compared with the preceding 2 months. Pediatric residents experienced the same effect, however, with significantly fewer reported medical errors in August. The reasons for this were not explored in this initiative; however, we postulate that the hospital had more reported medical errors in July due to mistakes by the influx of new doctors and increased responsibility of trainees promoted to the next level of training.

The “July Effect” is defined as the patients’ *experience* of more adverse events due to the uptick in new trainee medical errors.^1^ In our 5-month span evaluation with our methodology, it is difficult to determine a true “July Effect.” However, our investigation does show that the rate of adverse patient events due to reported trainee medical errors was rare and not significantly different by month.

The discrepancy between reported increased medical errors and the absence of increased adverse events from those errors could be explained by increased vigilance and concern from multiple levels of front-line staff including nurses, respiratory therapists, pharmacists, and supervising physicians in July. This involvement of staff can prevent trainee medical errors reaching the patient to become an adverse event. However, there is another possibility for this discrepancy, which leads to the most significant limitation of this report. Specifically, more error reporting could have occurred in July, given a heightened awareness of new physicians by supervisors and ancillary staff at the hospital as opposed to July truly having more trainee medical errors. July had the second lowest number of incident reports in our time frame but the highest proportion of pediatric trainee–reported medical errors per total incident reports. The higher proportion means that there still could have been hypervigilance among staff regarding pediatric trainees, which could have translated into more incident reports specific to trainees while overall incident reports were lower.

Our investigation has other limitations as well. First, incident reporting is voluntary and subject to its own inherent limitations. Some departments may report more and others less. Another limitation is that the data rely on the reviewers to determine trainee involvement and adverse events. There was no a priori calibration testing of assessing medical errors and adverse patient events by the reviewers. To enhance reliability, however, there were 3 separate independent reviewers of each error. An additional limitation is that we queried a 5-month snapshot as opposed to a full year. The rationale was that the 2 months before and after July would offer the most consistent patient population, whereas winter months may add confounding effects such as varying diagnoses and patient volume influences.

Interestingly, we noticed a rise in reported trainee medical errors in September similar to July. We do not have an explanation for this trend. One potential possibility is that in September pediatric trainees have increased autonomy and decision making with less stringent supervision leading to increased reported medical errors. However, more research is needed to verify a September uptick.

## CONCLUSIONS

In this single-center evaluation at a free-standing tertiary care academic medical center, hospital data suggest a spike in pediatric trainee–reported medical errors in July but not in adverse patient events from those errors. Further hospital-based investigations that use rigorous, sensitive data collection methods over the course of multiple years should be employed to confirm that a “July Effect” exists. We suggest that future studies explore factors present in the supervisory environment in July that may prevent progression to adverse events.

## ACKNOWLEDGMENTS

The authors thank Dr. Dewesh Agrawal and Lisa Scafidi from Children’s National Health System for their valuable input and thoughts in understanding and evaluating the “July Effect.”

## DISCLOSURE

The authors have no financial interest to declare in relation to the content of this article.
